# Using Participatory Spatial Tools to Unravel Community Perceptions of Land-Use Dynamics in a Mine-Expanding Landscape in Ghana

**DOI:** 10.1007/s00267-021-01494-7

**Published:** 2021-07-01

**Authors:** Jane J. Aggrey, Mirjam A. F. Ros-Tonen, Kwabena O. Asubonteng

**Affiliations:** 1 University of Amsterdam, Amsterdam Institute of Social Science Research (AISSR), Nieuwe Achtergracht 166, 1018 VW Amsterdam, The Netherlands; 2 United Nations University Institute for Natural Resources in Africa (UNU-INRA), International House, Annie Jiage Road, University of Ghana, Legon Campus, Accra, Ghana

**Keywords:** Participatory mapping, Artisanal/small-scale gold mining (ASM), Food cropping, Landscape configuration, Inclusive landscape governance, Ghana

## Abstract

Artisanal and small-scale mining (ASM) in sub-Saharan Africa creates considerable dynamics in rural landscapes. Many studies addressed the adverse effects of mining, but few studies use participatory spatial tools to assess the effects on land use. Hence, this paper takes an actor perspective to analyze how communities in a mixed farming-mining area in Ghana’s Eastern Region perceive the spatial dynamics of ASM and its effects on land for farming and food production from past (1986) to present (2018) and toward the future (2035). Participatory maps show how participants visualize the transformation of food-crop areas into small- and large-scale mining, tree crops, and settlement in all the communities between 1986 and 2018 and foresee these trends to continue in the future (2035). Participants also observe how a mosaic landscape shifts toward a segregated landscape, with simultaneous fragmentation of their farming land due to ASM. Further segregation is expected in the future, with attribution to the expansion of settlements being an unexpected outcome. Although participants expect adverse effects on the future availability of food-crop land, no firm conclusions can be drawn about the anticipated effect on food availability. The paper argues that, if responsibly applied and used to reveal community perspectives and concerns about landscape dynamics, participatory mapping can help raise awareness of the need for collective action and contribute to more inclusive landscape governance. These findings contribute to debates on the operationalization of integrated and inclusive landscape approaches and governance, particularly in areas with pervasive impacts of ASM.

## Introduction

Reconciling global and local needs for sustainable food and energy production while achieving poverty reduction, biodiversity conservation, and climate resilience are key challenges in multifunctional landscapes (Sayer et al. 2013; Milder et al. 2014). Landscapes are continuously changing, particularly when new livelihood activities are introduced. One such cause of landscape dynamics throughout the Global South is the mining industry, specifically the proliferation of artisanal and small-scale mining (ASM) (Cuvelier 2019; Verbrugge and Geenen 2019). Ghana is no exception, and extensive literature exists on both the expansion and effects of mining (e.g., Antwi et al. 2017; Pijpers et al. 2020) and the associated land-use dynamics (e.g., Basommi et al. 2016; Awotwi et al. 2018; Wu et al. 2019). These land-use changes may create synergies across livelihood activities (Banchirigah and Hilson 2010; Okoh and Hilson 2011; Hilson et al. 2013) and trade-offs (Cuba et al. 2014; Nyame and Grant 2014; Ferring and Hausermann 2019). Regarding the latter, specific concerns exist about the degradation of farming land and the disruption of local food systems (Botchwey et al. 2018; Hausermann and Ferring 2018; Kumah and Adum Nyarko 2018). Therefore, monitoring and assessing rural landscape change are essential for preventing such trade-offs and the governance of rural mining landscapes.

Many studies have assessed land-cover changes using remote sensing (e.g., Benefoh et al. 2018; Moomen and Yussif 2019; Obodai et al. 2019) or modeling (Awotwi et al. 2018). Seeking to explain the underlining causes of observed patterns, practitioners increasingly resort to contextually embedded knowledge (van Ewijk and Baud 2009; Pfeffer et al. 2013). In this paper, we define contextually embedded knowledge as all forms of non-codified (generalized/scientific) knowledge, including tacit practice-based knowledge, technical expert knowledge from experience, and contextual cultural knowledge (‘the way of doing things’) (van Ewijk and Baud 2009). In doing so, we acknowledge that rural people are custodians of locally embedded knowledge about their environment and associated problems and capable of providing and suggesting possible solutions (see also Somuah et al. 2021 and Asubonteng et al. 2021, this issue). Mapping (‘spatializing’) and collectivizing such knowledge can be an important means to uncover people’s perspectives of landscape change. As such, it creates a basis for awareness-raising, collective action, empowerment, and inclusive landscape governance that takes views of local inhabitants into account (Pfeffer et al. 2013; Somuah 2018; Asubonteng et al. 2021, this issue). Hence, this paper focuses on local spatial knowledge, which we define as place-based knowledge that people acquired through their long-standing relationship with the landscape where they live and work (McCall 2021). Such knowledge is best gathered through participatory approaches (IFAD 2009).

Participatory mapping has emerged as a powerful example of participatory approaches for development with prospects of empowering marginalized groups (McCall and Minang 2005; Chambers 2006; Sletto 2009). It encompasses “accessible and free-ranging visual methods in an individual or group interview setting to interrogate qualitative research questions” (Emmel 2008, p. 1). Participatory mapping entails the visual representation on maps of all resources and socio-cultural and natural physical features that community members identify as part of their environment, backed by a story (IFAD 2009). However, certain features are sometimes deliberately left out to obscure their presence for outsiders (Somuah 2018; McCall 2021). Mapping potentially excites the interest of community members in pressing land issues, leading to inclusive decision-making (IFAD 2009; Sletto 2009).

However, studies using perception-based landscape change assessment are scarce (but see Asubonteng et al. 2021, and Somuah 2021, this issue). To our knowledge, this paper is among the few that employ participatory mapping to investigate landscape changes across time in a mine-expanding landscape from the perspective of landscape users (i.e., small-scale farmers and miners). We chose this approach to trigger participants’ reflection on the adverse effects of past and future land-cover and land-use changes. Hence, the objective of this paper is twofold. First, we aim to unravel how communities in mine-expanding landscapes perceive the spatial dynamics of their landscapes and the implications thereof, notably on land available for food production. Second, by taking an actor perspective to the analysis, we aim to contribute to the debate on how participatory spatial tools such as participatory mapping can stimulate landscape actors such as small-scale farmers and miners to take a proactive stand in landscape governance.

After providing context on Ghana’s bifurcated mining sector, we present the research methodology with a description of the study area and data collection and mapping methods. In the results section, we analyze the perceptions of landscape dynamics in six communities based on participatory maps of landscape composition and configuration in the past (1986), present (2018), and anticipated future (2035). In the discussion, we explain the observed spatial trends and deliberate on the value of participatory mapping for inclusive landscape governance. Regarding the latter, we argue that participatory mapping helps trigger participants’ insights into the potential effects of landscape change and how adverse outcomes in the future can be avoided. The concluding section answers the research question, highlights implications, and formulates policy recommendations and suggestions for future research.

## Ghana’s Mining Sector

Ghana is rich in mineral resources, including gold, diamonds, bauxite, manganese, and—more recently—oil and gas. Dating back to the 4^th^ century (Gbireh et al. 2009), gold mining has a long and important history in the county, especially in the Birimian and Takwain gold belts in the Ashanti, Western and Central Regions (Hilson 2002; Smith et al. 2016). In 2019, the mining sector contributed 4.5% to the country’s real gross domestic product (GSS 2018). With minerals accounting for the highest gross merchandised receipt of 43%, gold is Ghana’s leading contributor with export receipts of USD 6.230 billion in 2019 (The Ghana Chamber of Mines 2019). The sector provided direct employment for 42,576 people in 2015 (GSS 2015).

Ghana knows two distinct types of mining operations: large-scale and artisanal, and small-scale mining (ASM). There were 13 large-scale mining companies in 2015 (Minerals Commission 2015), and an estimated 1350-1400 licensed ASM operations in the country in 2017 (Hilson 2017). ASM is a form of mining that uses simple machinery to mine precious minerals (Ofosu-Mensah 2016). In Ghana, ASM focuses mainly on gold production, and it is currently the only way of sourcing diamonds (Bansah et al. 2018). In addition to the registered ASM operations, there are unlicensed operations, colloquially referred to as *galamsey* (ibid). There is no distinct difference between registered and unregistered ASM miners regarding their organization and technology, except that registered ASM operators have secure land tenure rights (Teschner 2012; Ofosu-Mensah 2016). In the past, ASM operations mainly employed simple tools such as head pans, pickaxes, and shovels to mine gold in circular dug pits called mine pits, with an approximate diameter of 1 m and measuring several meters in depth (Kyeremateng-Amoah and Clarke 2015). However, under the influence of Chinese miners, the activity is becoming more sophisticated with heavy machineries such as trommels, changfans, excavators and bulldozers, and varying forms of labor organization (Ferring et al. 2016; Yankson and Gough 2019). ASM accounts for more than 30% of the country’s gold output and provides livelihoods in areas with few or no alternative employment opportunities (Bansah et al. 2018). The sector employs over 200,000 people, of whom an estimated 85% operate illegally (Eduful et al. 2020).

Before the 1980s, ASM was unregulated and received almost no government support (Ofosu-Mensah 2016). However, the Small-Scale Gold Mining Law (PNDCL 218) was passed in 1989 to legalize the activity, and the Mercury Law (PNDCL 217) and Precious Minerals and Marketing Corporation Law (PNDCL 219) were put in place to formalize the sector (Hilson et al. 2007). High registration fees and bureaucratic processes threatened the formalization process, producing a semi-formal sector that operates with different degrees of legal documentation (Teschner 2012). Due to mercury use and continuous pitting and trenching, the ASM sector has adverse environmental effects on water and land in particular (Clifford 2017; Kumah and Adum Nyarko 2018; Eduful et al. 2020). Moreover, concern exists about the degradation of farming land due to ASM operations and its implications for food production (Kumah and Adum Nyarko 2018; Ferring and Hausermann 2019; Eduful et al. 2020). Due to these challenges, ASM has been tagged as a ‘menace’ that has to be fought by the government (Hilson 2017), resulting in a ban and a military-style approach in recent years with limited success (Eduful et al. 2020; Hilson and Maconachie 2020; Osei et al. 2021).

This paper focuses mainly on the spatial implications of expanding ASM operations in rural areas from a community perspective, recognizing that ASM and farming are intricately entwined (Maconachie and Hilson 2011; Ofosu-Mensah 2016; Mkodzongi and Spiegel 2019). ASM is an economically powerful livelihood option in rural areas, supporting households’ livelihoods and threatening farming land and forests on mineral-rich lands. Because of this, mining in agricultural and forest landscapes is an issue of great scholarly and policy concern in an era when forests have become essential in fighting climate change and concerns exist about how to ensure food security of a growing population (Hirons et al. 2014; Ickowitz et al. 2014; Hirons 2015).

## Methodology

### Study Area

**Fig. 1:**
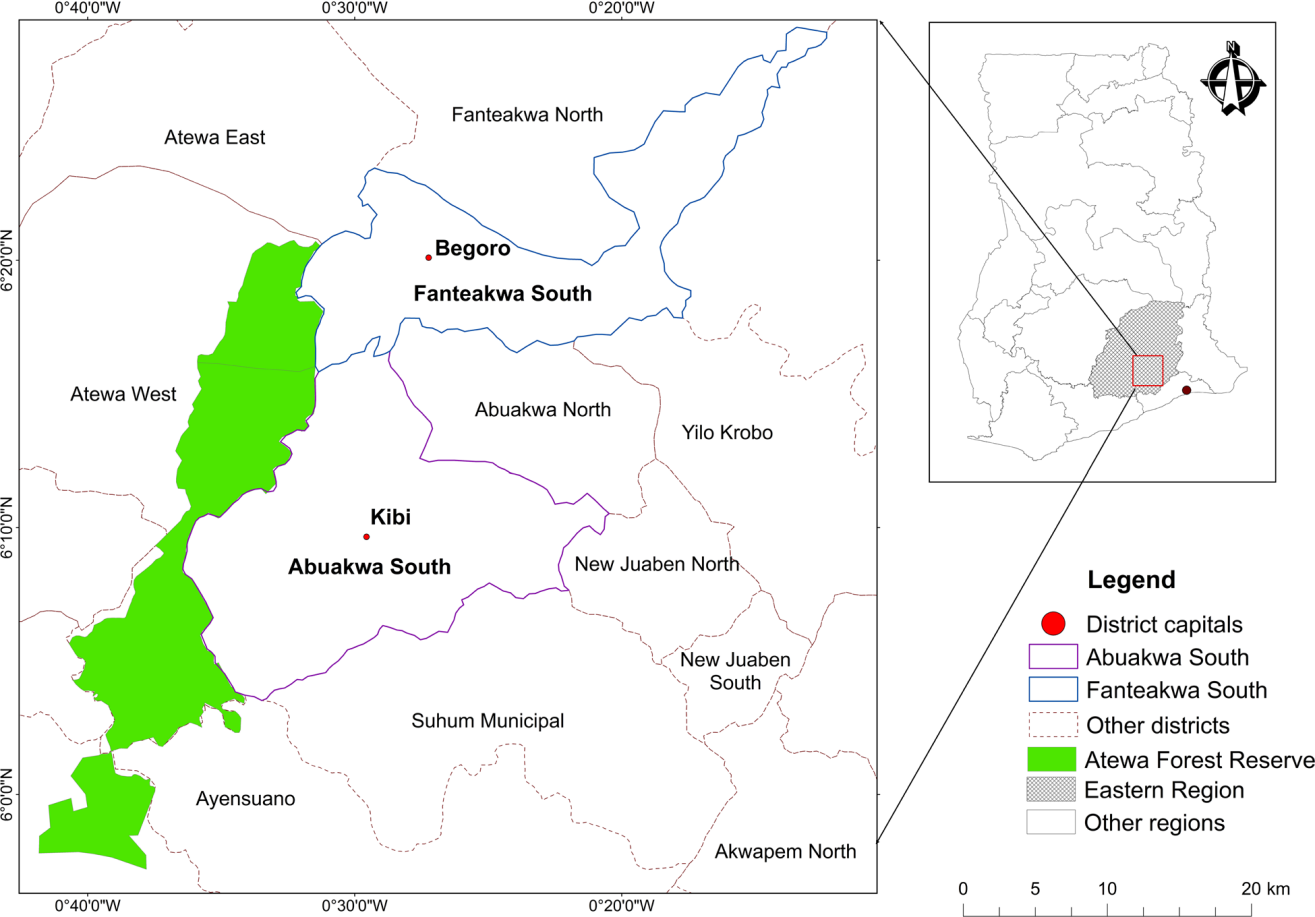
Map of Abuakwa South Municipality and Fanteakwa South District in the Eastern Region of Ghana (*Source:* Adapted from https://data.humdata.org/dataset/ghana-administrative-boundaries). NB The actual community names and their geographical locations are not shown to honor the anonymity agreement between the researchers and respondents

The study area covers two administrative districts in the Eastern Region of Ghana: Abuakwa South Municipality and Fanteakwa South District (Fig. 1). The area is located in the moist semi-deciduous southeast subtype ecological zone (Hall and Swaine 1981). The mean annual rainfall of 2,000 mm falls in a bimodal pattern with a peak from May to June and September to October. The mean annual temperature is 30 ^o^C (Fanteakwa South District Assembly; Abuakwa South Municipality Assembly 2018). The landscape is well-drained by rivers, several of which are seasonal and flow from the Atewa Forest Reserve, which borders the landscape on the eastern side. The region has fertile lands where staple foods (mainly plantain, maize, cocoyam, cassava, and vegetables) and tree crops (cocoa and oil palm) are grown. The landscape falls within the country’s ‘food baskets’ (Hilson and Garforth 2013). The area is endowed with mineral deposits such as bauxite, kaolin, and alluvial gold, which has attracted many miners (RMSC 2016). Because of the agricultural activities, mineral deposits, and the prevalence of ASM (both legal and illegal), this area was selected for this study on the perceived effects of ASM on a mixed farming-mining landscape.

### Sampling

Six communities were purposively selected from the Abuakwa South Municipality and Fanteakwa South District, based on varying proportions of farming and ASM in the landscape. Communities were categorized to distinguish between high farming-low mining, low farming-high mining, and high farming-high mining communities, based on RMSC (2016), which mapped mining activities in the environs of the Atewa Forest Reserve. From each category, two communities were selected where participants actively engaged in farming, ASM, or both (Table 1). Other selection criteria included the willingness of chiefs and communities to participate in the study, accessibility, and safety considerations. To protect the safety and anonymity of communities and participants, we anonymized the selected communities by using the pseudonyms presented in Table 1.

**Table 1:** Selected communities in the Abuakwa South Municipality and Fanteakwa South District based on the relative prevalence of farming and mining (*Source:* Adapted from RMSC 2016, unpublished)

	ASM	
Farming	High	Low
High	Gyesame^a^	Mudawka^a^
	Nanaase^b^	Osau^b^
Low	Makisa^a^	–
	Wanoiso^b^	

^a^Abuakwa South

^b^Fanteakwa South

Participants in the mapping exercise were selected in collaboration with community leaders, based on (i) whether they had lived long enough in the community to have witnessed significant changes in the landscape, (ii) their knowledge of the landscape, and (iii) involvement in ASM and/or farming in the past and/or present. The age range of participants was between 40 and 75 years—with those closer to 40 giving more input on recent changes and current features of the landscape. The mapping exercise, held in 2019, was done with eight members from each community, with equal gender representation where possible. The number of participants was limited to eight per community for easy group management, team building, and time and cost-effectiveness.

### Uncovering Landscape Dynamics

The study employed elements from PROFOR toolkit 3—timelines and trends (see Shepherd and Blockhus 2007)—before starting the mapping exercise to trigger participants’ thoughts and understanding of land-cover and land-use change across time. In focus group discussions, participants agreed on the main land-cover and land-use types in each community: food- and tree-crop lands, settlement, mining sites, forest, and water bodies. Participants then discussed the prevalence and status of these landscape components and the natural and anthropogenic drivers causing changes. This was done to help visualize and map the land-cover and land-use types for the past and present. A future landscape scenario based on business-as-usual was also discussed. Considerations in determining the start of the timeline were major past events and respondents’ memory span over time. Participants agreed that 1986 would be a good starting point, as it was the year in which the landscape had recovered from natural bushfires that hit the country in 1983. The year used for the present situation was 2018, which was the immediate past before the mapping exercise. The year 2035 was selected for the future landscape scenario because it represented a foreseeable future for most participants. Participants discussed the resources and land uses present in the landscape at the three time points and the causes of the changes. This discussion helped visualize landscape composition and configuration at the three time points to uncover dynamics along the selected timeline (1986–2035).

### Visualizing the Land Cover and Land Use

Land cover and land use were mapped using participatory mapping. Tools used for the mapping exercise included markers, pencils, erasers, a 2017 Google Earth (high-resolution) image of each community, and a base map^1^ of each community digitized from the 2017 Google Earth image using ArcGIS software and printed on A0 paper. The base maps comprised of roads extracted from Google Earth and water bodies and forests drawn from national shapefiles (Ghana at a glance, EPA), with only the base map for the present showing the extent of settlement area digitized from the Google image. The Google image and base map of the community helped participants to orient themselves geographically. All participatory maps were drawn on a foldable table, which stood at breast height for ergonomic reasons. The mapping started with explaining what community mapping is, the techniques involved, and how to read a map. Training of participants was crucial to ensure a collective understanding of the mapping process. For ethical reasons and to gain trust, the purpose, time needed, ownership of outputs, and the sketching itself (names of features and symbols used for them) were discussed in detail (Rambaldi et al. 2006a and b; Emmel 2008; Verplanke et al. 2016). Considering the ongoing ban on ASM and military-style government interventions to combat illegal mining, safety issues also required explicit attention to take away any feeling of insecurity.

As the participants were most familiar with the current landscape, this was the first to be sketched after the participants had familiarized themselves with the 2017 Google image and the base map of their landscape. First, they mapped the landmarks that were not visible on the base map. The base map was then divided into four, and in each portion, a river and a major road were used as reference points for landscape features identified in the plenary discussions. Participants were asked to go back in time to map the past landscape when it was recovering from the 1983 nationwide bushfires (a historical event well marked in the minds of many). Based on recall, discussions on timeline events, and using the map of the current landscape as a benchmark, participants mapped the settlement boundary and landmarks present in 1986 and subsequently populated the map using the approach used for the 2018 map (present). Building on the current landscape and assuming a business-as-usual scenario, participants then mapped their future landscape, subsequently discussing the effects of the changes in land use.

A total of 18 participatory maps were produced; three for each community for the past (1986), present (2018), and the anticipated future (2035), respectively. The latter map represents participants’ forecasts based on current trends and policies. Each set of maps reveals trends in land cover and land uses. During validation meetings in each community, all inhabitants were allowed to give inputs to the maps, and the consequences of land-use changes were discussed. This meeting was usually organized on a taboo day—a day specific for each community on which farming, forest, and fishing activities are prohibited.^2^ All discussions during the mapping process and map validation were recorded with the permission of the participants.

### Processing the Maps

The participatory maps were scanned into digital format for legend harmonization. Scanned maps were imported into ArcGIS version 10.6 for manual digitizing of mapped features into shapefiles. A standard and appropriate symbology was adopted for all maps, imitating ground features to make it easier to relate the map to features in the real world. Recomposing the maps ensured a clear delineation of features, and the common legend allowed for the comparison of individual maps across years and communities. The audio recordings of the negotiations and discussions from the mapping process were repeatedly listened to, from which we took note of the reasoning behind perceived changes in the landscape.

The community perceptions of the spatial dynamics in the six landscapes across time were analyzed in two parts. First, we analyzed perceived changes in the land-cover types mapped and their relative sizes (composition). Second, we analyzed the perceived changes in the spatial arrangement (configuration) of the land-cover types and the degree of landscape integration (heterogeneity) and segregation (homogeneity).

## Results

Below we present the results of the participatory mapping for Abuakwa South Municipality and Fanteakwa South District, respectively, in the order of communities characterized by high farming-high mining, low farming-high mining, and high farming-low mining (Table 1).

### Dynamics in Abuakwa South Municipality

#### The Gyesame Landscape

**Fig. 2:**
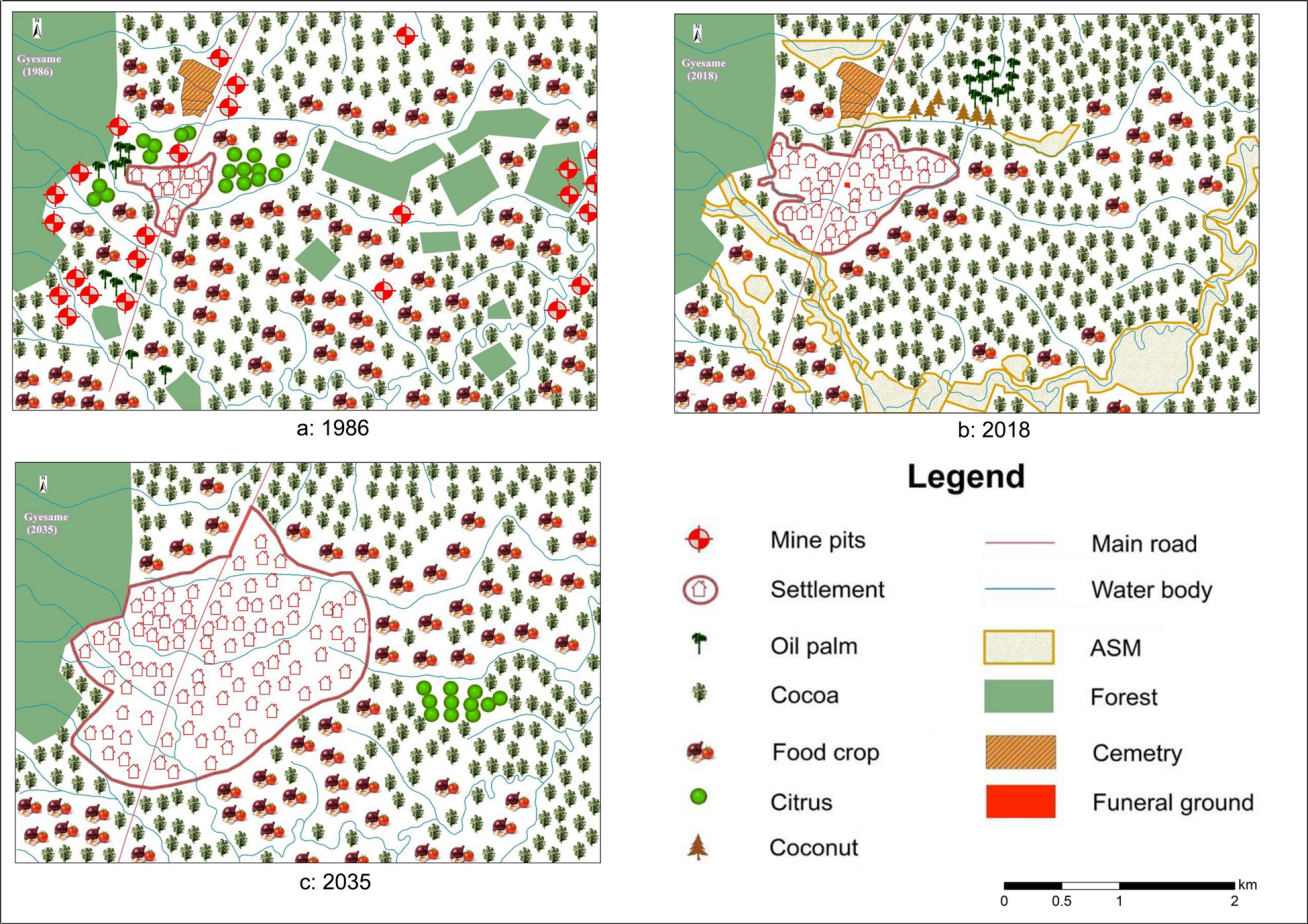
Participatory maps of the Gyesame landscape in 1986, 2018 and 2035^3^

In terms of composition, the participatory maps of the Gyesame landscape show a predominantly agrarian landscape despite the occurrence of ASM activities in the present and anticipated settlement expansion in the future (Fig. 2a–c). Past mining is mapped as mine pits along water bodies and a major road and in farms in the West (Fig. 2a). These are replaced with ASM in substantial areas along water bodies in the South in the map depicting the present (Fig. 2b). Food crops and cocoa are the main crops in all maps, causing significant deforestation from the past to the present, as no forest outside the forest reserve in the East is mapped in the present. Food crops are mapped along water bodies in the past and in concentrated patches in the western part of the present landscape but have disappeared from the center area where cocoa has expanded. Participants expect this to recover and foresee a mixed cocoa-food crop landscape again for the future (Fig. 2c). Citrus is not mapped in the present (Fig. 2b) but is anticipated to reoccur in the future, while the isolated patches of oil palm in the West (Fig. 2b) disappear in the future (Fig. 2c). Unlike the other crops, coconut was mapped only in 2018. The maps show continuous settlement expansion into farmlands, with participants predicting expansion up to the borders of the forest reserve on the western side. The forest reserve in the West and water bodies and roads remain stable features in the landscape.

In configurational terms, the maps suggest that the Gyesame landscape has moved toward a segregated landscape. Whereas the mapped past landscape shows mixed land use, the present map shows increasing homogenization (Fig. 2b). Different land-cover types are mapped as distinct features, with farmlands and settlement areas being the dominant features. Forests mapped as patches surrounded by food crops and cocoa in the past (Fig. 2a) are converted to cocoa farms in the present, and ASM has replaced food crops and some cocoa in the South (Fig. 2b). The anticipated future shows previously mined lands along water bodies being mapped as food-crop lands again (Fig. 2c), with cocoa dominating in-land and a somewhat less segregated landscape than the present, as food crops and cocoa are mapped in an alternating pattern. Selected as a high farming-high mining landscape based on RMSC (2016) (Table 1), the participatory maps rather reflect an evolution from very high farming-very low mining-very low settlement in the past to high farming-low mining-very low settlement in the present. The expectation is that the landscape will move toward high farming-no mining with moderate settlement cover in the future (see Fig. S1 and accompanying text in the supplementary material for the method used for the landscape categorization).

#### The Makisa landscape

**Fig. 3:**
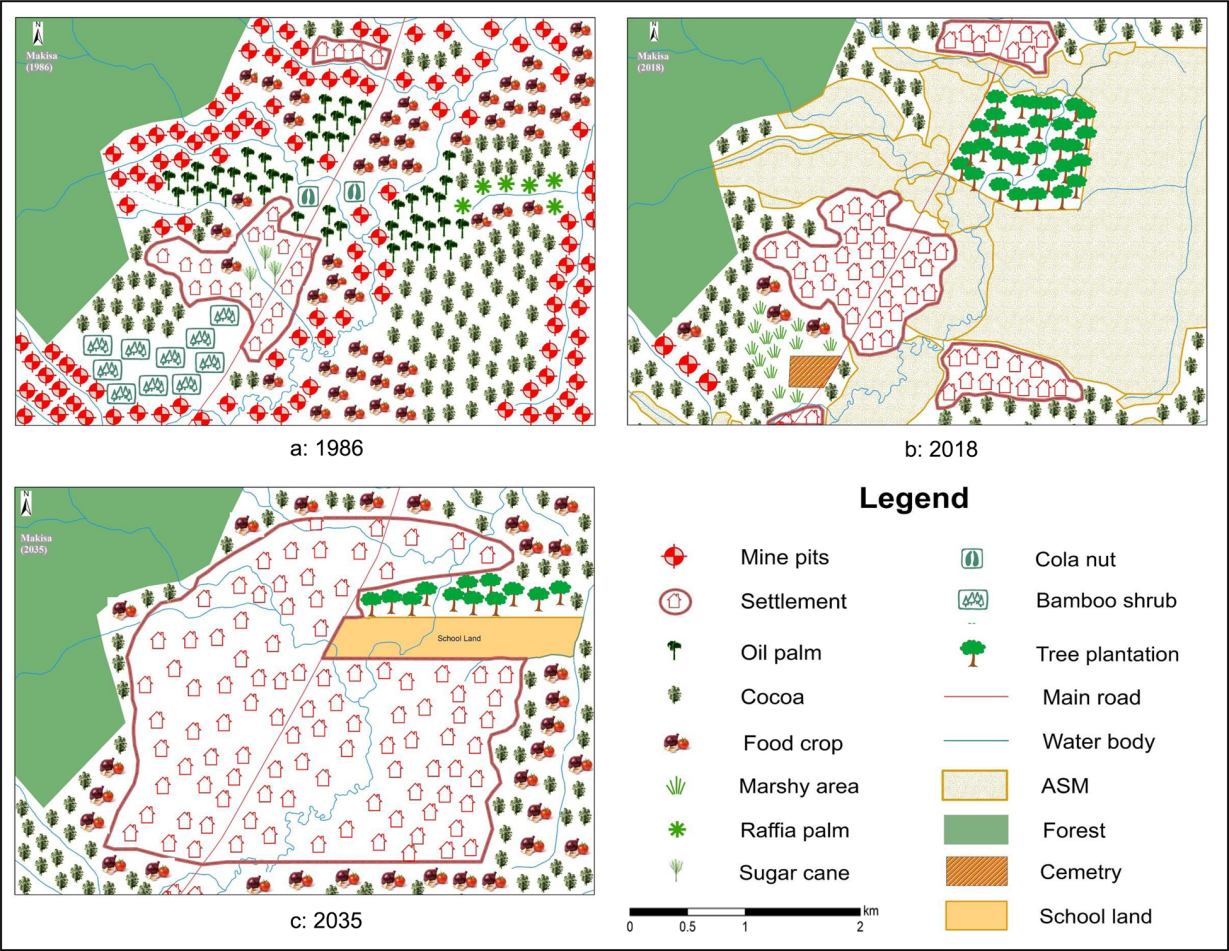
Participatory maps of the Makisa landscape in 1986, 2018 and 2035

In the Makisa landscape, the mine pits scattered over the landscape in the vicinity of rivers on farming land with food crops and cocoa are evidence of past mining activities (Fig. 3a). Mine pits are associated with ASM and mapped as such in the current landscape (Fig. 3b). They are expected to disappear in the future. A stretch of raffia palm along a river in the mid-east has given way to ASM and is expected to be school land in the future (Fig. 3c). Changes in the composition of land-cover types reveal that crops grown in the past were mainly food crops and cocoa. Sugar cane and raffia palm, which occurred naturally, and the non-timber forest products cola nut and bamboo no longer recur on the maps for the present and future. In 2035, food and cocoa are expected to grow on previously mined lands. A forest reserve in the Northwest remains a stable feature in the landscape. At the same time, a private timber plantation appears in the present landscape and is expected to extend further toward the East but will lose its southern half of the area to school land.

This school land is expected to cover previously mined lands stretching from a major road to a boundary river on the east. A stand of bamboo shrub in the southwestern portion of the 1986 map (Fig. 3a) is replaced with the underlying marshy land in the present and expected to be part of the settlement area in 2035. The settlement area shows a trend of continuous expansion, taking up nearby farmlands in 2018 (Fig. 3b) and previously mined lands in the future, to become the dominant land cover in 2035 (Fig. 3c). Forest, water bodies, and roads remain fairly constant.

In terms of configuration, the past Makisa landscape was already mapped as somewhat segregated: mixed-use only occurred in the form of mine pits on farming land. In the present, this is more pronounced, with ASM, settlement, and the timber plantation mapped as dominant land uses, and a cocoa stand in the West. The cocoa stand in the Southwest is being interplanted with food crops. The mapped future shows a landscape dominated by a settlement surrounded by a timber plantation and mixed cocoa and food-crop patches. Participants position future food-crop patches on remaining lands around the settlement, including previously mined lands.

The maps suggest a shift from moderate farming-high mining-very low settlement in the past to very low farming-high mining-low settlement in the present, confirming the basis on which this community was selected (Table 1). This mine-dominated landscape is expected to move toward low farming-no mining outside the settlement area in the future, with some farms occurring on previously mined land and food and cocoa occurring in almost equal portions. Settlement cover is expected to be remarkably high in the future (Fig. S2, supplementary material).

#### The Mudawka landscape

**Fig. 4:**
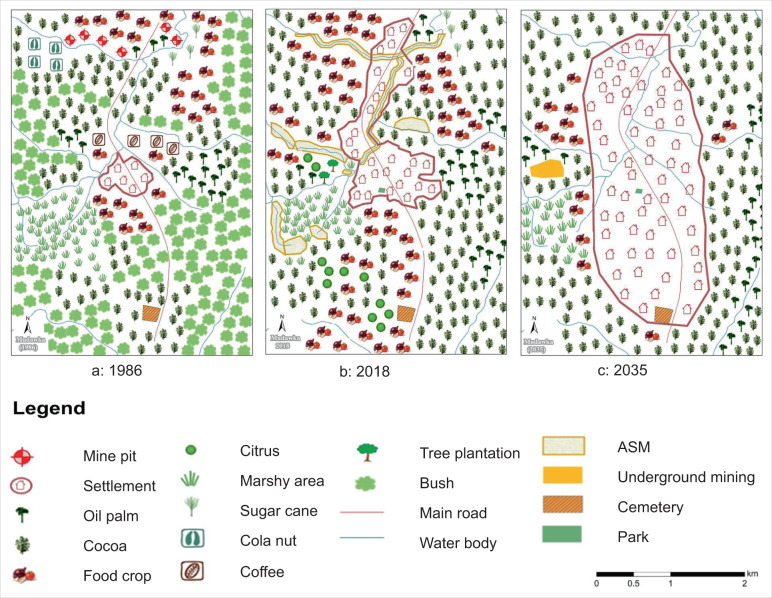
Participatory maps of the Mudawka landscape in 1986, 2018 and 2035

The participatory map of the past Mudawka landscape (Fig. 4a) shows mining pits in the North, which were not mapped for 2018. However, mining is widespread in the 2018 map as mining sites along water bodies, with portions of past marshy land converted to ASM (Fig. 4b). The map of the anticipated future landscape (Fig. 4c) shows that farmers expect ASM to disappear from the landscape and that mining sites will be converted to settlements and farms in 2035. The mapped future shows an underground large-scale mining site in the West, replacing citrus and food crops.

In terms of composition, the most notable change in Fig. 4a–c is how participants perceive and anticipate the expansion of the settlement. They also perceived the disappearance of crops such as sugar cane, cola nuts (*Cola nitida*), and coffee between the past and present. The maps further show how participants see the growing expansion of cocoa, mainly at the cost of forest and tree cover (‘bushes’) and land for food crops. The food-crop area has expanded from past to present, for instance, on previous marshy land. Still, farmers expect food-crop farming to drastically reduce in the future, primarily because they expect better and more secure incomes from cocoa production.

In terms of spatial configuration, the participants observe growing segregation in the landscape. Whereas the past landscape shows shaded cocoa in the East, after replacing bush with food crops and citrus in the present, the tendency is toward increasing homogenization with cocoa becoming dominant. This increasing segregation is also reflected in the dominance of the settlement and disappearance of bushy and fallow lands. While food crops were mapped as homogenous patches in the past, the map of the present shows more intercropping with cocoa, notably in the western portion of the landscape. While this intercropping remains in the Northwest, food crops disappear from cocoa-dominated areas in the East and Southwest in the mapped future. Instead, they appear in the marshy area and are further concentrated as a few patches along water streams and the southwestern border of the settlement (Fig. 4c).

Based on the relative proportions of land cover, the Mudawka landscape moved from low farming-very low mining-very low settlement in the past to high farming-low mining-low settlement in the present, the latter in line with Table 1. The landscape is expected to retain its agricultural features in the future, but like the Makisa landscape, settlement coverage is expected to increase substantially. Hence a new trend is anticipated for the future, with moderate farming, very low mining but high settlement coverage (Fig. S3, supplementary material).

### Dynamics in Fanteakwa South District

#### The Nanaase landscape

**Fig. 5:**
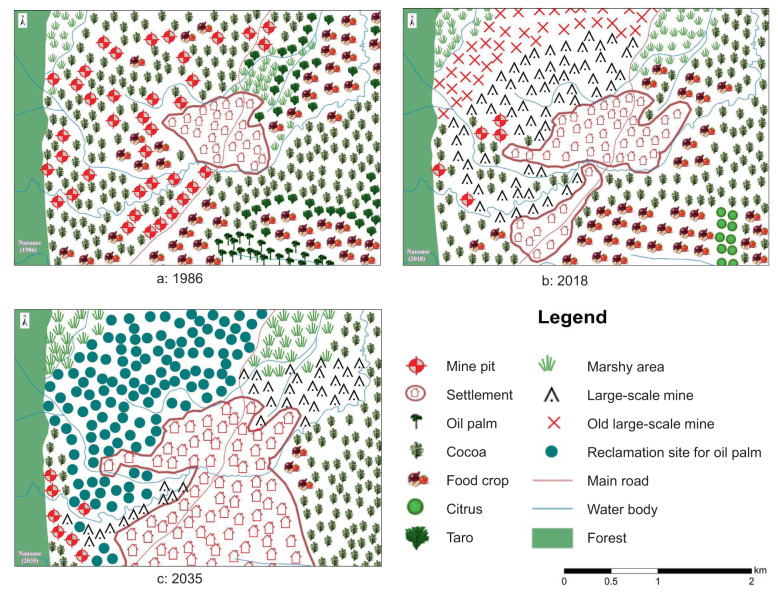
Participatory maps of the Nanaase landscape in 1986, 2018 and 2035

The maps of the Nanaase landscape show a mine-expanding landscape (Fig. 5a–c). Mining in the past is mapped as mine pits in farming land (Fig. 5a). The map of the present landscape reveals the dominance of large-scale mining (Fig. 5b), which is perceived to persist in the future (Fig. 5c). Food crops and cocoa appear in all maps but in decreasing sizes, while taro and oil palm mapped in the past (Fig. 5a) no longer appear on the maps of the present and future (Fig. 5b, c). Participants mapped citrus in the present, but these areas are expected to be converted to cocoa in the anticipated future. A unique feature in the map of the anticipated future is the substantial portion of reclamation sites for oil palm (Fig. 5c), which replaces most of the current large-scale mining area (Fig. 5b). The maps show that settlement is expanding southward at the cost of farming lands (cocoa and food crops). The forest that borders the landscape on the West, water bodies, and roads remain fairly constant.

The participatory maps of Nanaase show a shift from mixed land use in the past (Fig. 5a) to a highly segregated landscape in the future (Fig. 5c). The mine pits in farmlands and along water bodies and taro growing in marshy land are evidence of mixed land use in the past and present. Food-crop land is consistently mapped as homogenous stands: in the past west, south, and northeast of the settlement; in the present, more concentrated in the southern portion of the landscape, seemingly partly replacing cocoa. Food-crop land is virtually absent in the anticipated future, with a small portion remaining on the eastern side as an island surrounded by large-scale mining. In the present and anticipated future, mining and reclamation sites are concentrated on the landscape’s western side.

The maps of the Nanaase landscape suggest an evolution from a high farming-low mining-very low settlement landscape in the past to a moderate farming-moderate mining-low settlement landscape in the present (Fig. S4, supplementary material). The latter somewhat deviates from the typology in Table 1 as high farming-high mining. Participants mentioned that areas marked as reclamation sites in 2035 are to be reclaimed with oil palm. This reclamation is anticipated to increase areas under farming, which would push Nanaase toward a high farming-very low mining landscape in the future with moderate settlement coverage.

#### The Wanoiso landscape

**Fig. 6:**
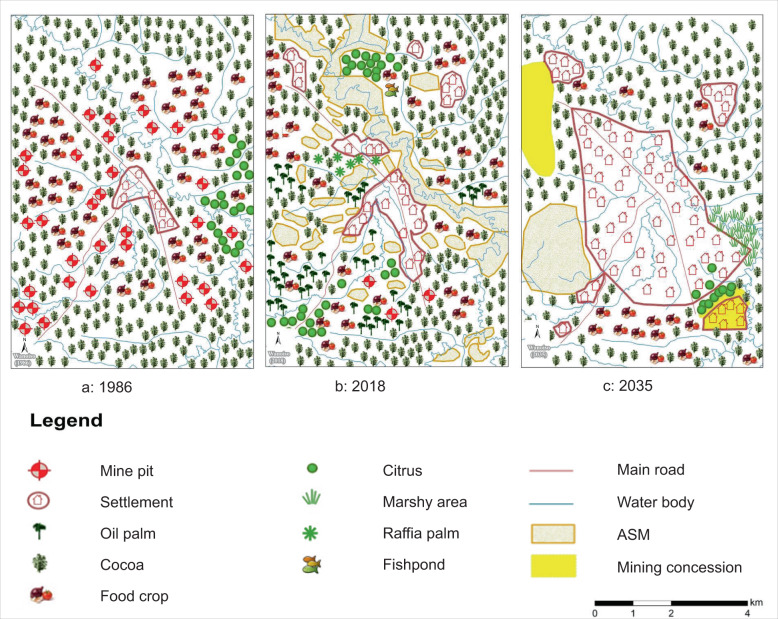
Participatory maps of the Wanoiso landscape in 1986, 2018 and 2035

Mining in the past Wanoiso landscape is mapped as mine pits in food-crop land (Fig. 6a), with most of them converted to ASM sites in the present (Fig. 6b). No traces of ASM are mapped for the future, but a mining concession is expected instead (Fig. 6c). Participants expect mined areas and farmland on the southeastern side in 2018 to be converted to marshy land in 2035 due to the prevalence of water bodies and the effects of mining. Cocoa, citrus, and food crops remain constant in the landscape, while a fishpond, raffia, and oil palm are mapped only in 2018 (Fig. 6b). The anticipated absence of oil and raffia palm in the future is attributed to the expansion of mining and settlement (Fig. 6c). Water bodies and roads remain relatively unchanged.

Regarding spatial configuration, participants mapped the past as a mixed land-use landscape, with mine pits in farmland and food crops in cocoa farms, and food crops mapped closer to the settlement than cocoa (Fig. 6a). In the inhabitants’ perceptions, the Wanoiso landscape is moving toward a segregated landscape with the spatial impact of ASM seen along water bodies and farmland. Significant portions of farming land in the present landscape are mapped as mining sites (Fig. 6b). The anticipated future landscape is entirely segregated and dominated by the expanding settlement and mining sites and concessions, with farmland substantially reduced and appearing only in the North (cocoa) and Southwest (food crops) (Fig. 6c). Remarkably, future food crops and citrus cultivation only appear in and around the mining concession in the Southeast. Citrus appears consistently across the three periods, but its location changes from predominantly in the East in the past to patches in the Southwest and center North in the present and a patch in the Southeast in the anticipated future.

From a high farming-low mining-very low settlement landscape in the past, the present Wanoiso landscape is seen as a mine-expanding landscape with moderate farming-moderate mining and is expected to transform into a moderate farming-low mining-high settlement landscape in the future (Fig. S5, supplementary material). The community perception of the landscape differs from the low farming-high mining characterization on the basis of which this landscape was selected (Table 1).

#### The Osau landscape

**Fig. 7:**
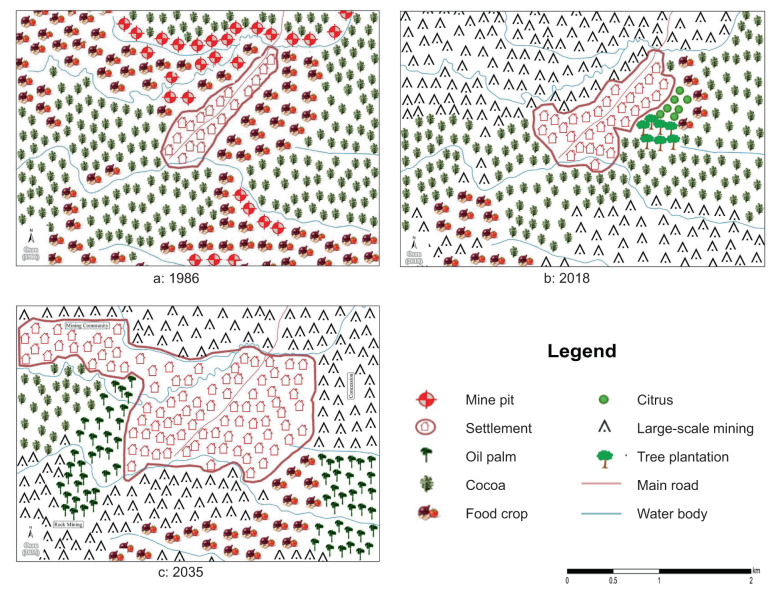
Participatory maps of the Osau landscape in 1986, 2018 and 2035

Like Nanaase, Osau is a mine-expanding landscape. Mining in the past landscape was mapped as mine pits located in food-crop land along water bodies (Fig. 7a). In the present, mining occurs on a large scale and is the prominent land cover (Fig. 7b). Large-scale mining is perceived to be significant in the future landscape, as a concession in the Northeast and as hard-rock mining in the Southwest.

Regarding composition, food crops and cocoa are mapped as the dominant crops in the past and present, with citrus and a tree plantation appearing in 2018 (Fig. 7b). The latter two are expected to be converted to settlement and large-scale mining in 2035 (Fig. 7c). In 2035, a mining settlement is mapped separately from the main settlement area. That makes settlement areas, together with large-scale mining, the dominant land cover in the anticipated future. Oil palm appears on previously mined lands in the West as a result of reclamation. Water bodies and roads remain fairly unchanged.

In terms of configuration, the participatory maps reveal mixed land use in the past, as evidenced by mine pits in farming lands and food crops in cocoa farms. However, cocoa and food crops are mapped separately, suggesting some degree of segregation. The maps of the present and anticipated future show a highly segregated landscape. In the present, farmland mapped on the western side appears as islands due to large-scale mining around them. Food crops in the present are mapped as a small patch northwest of the settlement (next to a timber and citrus plantation). Their area expands again in the anticipated future, notably in the South and Southeast.

The Osau landscape belonged to the high farming-low mining-very low settlement category in the past and has changed to a moderate farming-high mining-low settlement landscape in the present, contrasting the classification in Table 1 as high farming-low mining. In the anticipated future landscape, large-scale mining remains invariably high, while farming is expected to further decrease and the settlement to expand. Hence the qualification as a low farming-high mining-moderate settlement landscape in the future (Fig. S6, supplementary material).

### Explaining the Trends and Discussing Effects

In 1986, farming lands dominated the landscape. Their dominance in that particular year is an after-effect of an intense famine caused by a prolonged drought and a nationwide bushfire in 1983 (Dei 1988; Arthur and Arthur 2011). The fires had widely cleared the land from its vegetation, facilitating its preparation, while the return of rains and extra labor from Ghanaian returning from Nigeria facilitated the expansion of food-crop land (Asante et al. 2017).

‘After the 83 bush fires, food became abundant because there was no need for land clearing and preparation. You just planted, so a lot of people went into farming’ (Workshop participant Makisa, June 2019).

In the past, mining was an insignificant land use due to simple tools, low capital and technology investments, and low production and efficiency levels. Artisanal mining did not allow for massive gold exploitation because it required several months to dig deep and accumulate gold-bearing rocks without guarantees of finding deposits. As a research participant explained:

‘*Galamsey* became well known in the community in the late 1980s, but it was not like what we see now. It was [done by] individuals using their shovels, head pans, and pickaxe to look for gold in old mine pits’ (Workshop participant Nanaase, May 2019).

The stretch of the Atewa forest reserve that bordered some communities (Figs. 2, 3, and 5) remained a permanent feature in the landscape due to its fully protected status as a so-called Globally Significant Biodiversity Area (Weber and Fahr 2007; see also Somuah et al. 2021 this issue).

In the 2018 maps, both ASM and large-scale mining have become a prominent feature. The mining activities are mapped in places where old mine pits exist, suggesting that the latter are used as ‘gold trackers’. This is based on the general belief that the old mine pit system of gold mining was unable to exhaust all deposits:

‘The *galamsey* people continue with what our grandfathers did. They look for remnant gold in the old mine pits found in farming land and clear most lands with these old mine pits because they know that there is gold wherever these old pits are. The *galamsey* method could not have taken all the gold in the ground’ (Workshop participant Nanaase, May 2019).

Mining, food cropping, and settlements compete for space mainly along water bodies. Rain-fed food-crop farming is mainly done along water bodies because it facilitates easy watering (Kyei-Baffour and Ofori 2007). Many of these food-crop lands are transformed into mining sites because of the alluvial nature of gold deposits. Moreover, farmers prefer giving up their food-crop lands for mining rather than their cash-crop land (reflected in Figs. 4a, 6a, and 7a, but not 5a), due to the economic importance of the latter and—specifically in the case of cocoa—cultural attachment (Ataa-Asantewaa pers. comm., 2020).^4^ Farmers also consider the compensation package for damage to cash crops inadequate in most cases, while miners are somewhat cautious regarding mining in cocoa farms because compensation payments are higher than for food crops.

‘They (mining operators) paid GHS 1000 for an acre of a cocoa farm; GHS 900 to the farmer and GHS 100 to the chief, but for food cropland, they paid GHS 500, of which GHS 100 was given to the chief. Those who sold their lands lost their land and could not do anything with the meager amount they were given. I refused to sell my land, and I have been able to take my kids even to tertiary school’ (Workshop participant Nanaase, May 2019).

Another reason why farmers prefer food-crop land rather than cocoa to be converted to mining is government support for cocoa production. This includes seedling distribution, a free pest, and disease control program, a guaranteed price and market, the introduction of higher-yielding hybrid species, and improvements in road infrastructure in cocoa areas and marketing infrastructure (see also Laven and Boomsma 2012; Wessel and Quist-Wessel 2015). Cocoa farms are also regarded as property that can be used as collateral for credits (Wessel and Quist-Wessel 2015).

‘Now, a farmer can plant several acres of cocoa because we use weedicides and other farm inputs, making farming relatively easier. The government also supports cocoa farmers with inputs, and we sell our cocoa with almost no difficulty’ (Workshop participant Gyesame, May 2019).

This is not to say that no cocoa farms are converted to mining sites. It is worth the deal for both parties if gold deposits are promising.

Crops cultivated near mining sites are highly contaminated with mercury, lead, uranium, and arsenic, with detrimental effects on growth (e.g., Attiogbe et al. 2020). This—together with the conversion of marshy lands to mining sites—explains the disappearance of taro, bamboo, and sugar cane that occur naturally in these areas. Tree crops such as coffee (*Coffea* spp.) and cola (*Cola nitida*) also disappeared from the maps due to market failures. Although farmers combine cocoa with citrus to diversify their tree crops (Michel-Dounias et al. 2015), the occurrence of citrus is inconsistent across time, which can be attributed to pest and diseases as well as market failures (Brentu et al. 2012; Asare-Bediako et al. 2013; Asubonteng et al. 2021, this issue). Referring to oil palm, farmers explained that these were old stands that were tedious to maintain; new stands to be planted in reclamation sites were still to be planted.

The anticipated future landscape is dominated by settlement areas due to urbanization and infrastructure development, based on the expectation that mined lands are more suitable for infrastructure and settlement development than for farming. At the same time, some miners tend to invest their income in housing to sustain their wealth. Also, the on-site housing of large-scale mining workers in the Osau landscape is expected to contribute to settlement expansion. Despite the anticipated increase in food-crop area compared to the present, the future landscape is expected to face land scarcity for food cropping. The remaining fertile lands are destined for cocoa. Farmers expect to maximize the productivity of their food crops through intensification.

There are no mapped ASM sites in the future. Only large-scale mining of rock and underground mining is anticipated to occur in some areas. Participants explain this by the depletion of gold deposits that can be mined with ASM technology. Large-scale mining is anticipated to occur in already demarcated concessions, confirming reports that large-scale mining receives more government attention than the ASM sector (Banchirigah 2006).

Although the maps show a few oil palm stands in the future, in reality, these areas may become more prominent. Oil palm does well in poor soils and is, therefore, the preferred crop used in the reclamation of mined lands (pers. comm. Assistant Municipal Chief Executive April 2019; pers. comm. representative of the Okyehene Environmental Foundation, October 2019). The suitability of the area for oil palm is evidenced by land cover in the adjoining districts, where oil palm is the second major tree crop and appreciated by the farmers for generating a steady income (Asubonteng et al. 2018). However, participants in Nanaase have no hope in these reclamation efforts due to some unpleasant experiences:

‘You cannot do anything on the land they leave behind. They say they are doing reclamation. Go and see what they call reclamation. They say they are planting oil palm. It is nothing to be enthused about. Mining did not come to help us at all’ (Workshop participant Nanaase, May 2019).

The participants further discussed the consequences of landscape dynamics. First, they fear rising food prices due to the conversion of food-crop land to mining sites, cocoa farms, and settlements. Second, they are aware that the quality of food crops is compromised due to water and soil pollution caused by mining and excessive use of agrochemicals and tuber rot in cassava grown on mined land and near mining sites. Third, uncovered abandoned mine pits grown with weeds pose several health risks. They are death traps to farmers on their way to the farms, breeding grounds for mosquitoes, increasing malaria incidence in the communities, and hiding places for snakes. Fourth, the participants anticipate a declining farming population due to less availability of food-crop land. Moreover, the risks have made farming unattractive, particularly for the youth, who see better prospects in mining where they can make “quick money”. Fifth, the trend toward segregation was of concern to the study participants because of livelihood impacts associated with declined availability of non-timber forest products from forests and fallow land.

Last but not least, the mapping process and validation meeting provoked discussions on actions to be taken.

‘We as farmers should unite and not give our lands to miners. You think it is only your land you sold, forgetting that they (miners) will have to pass through your neighbors’ farms before they get to yours and even channel their wastewater through farms they have not purchased’ (Workshop participant Osau, May 2019).

‘It is about time we become inquisitive about everything that happens in our community and stop leaving everything in the hands of community leaders. We should question happenings in our community we do not understand and hold our community leaders accountable (Workshop participant Makisa, June 2019).

Hence, the mapping process triggered participants’ awareness of the need to engage and play a role in landscape governance.

## Discussion

### Competing land uses

Unlike studies that employ remote sensing to analyze dynamics in rural land cover and land use (e.g., Moomen and Dewan 2016; Benefoh et al. 2018; Moomen et al. 2019), this study has employed participatory mapping to uncover a community perspective of such dynamics. This resulted in maps revealing how community dwellers in two administrative areas in Ghana’s Eastern Region perceive changes in landscape composition and configuration across a timeline running for 1986–2035. The analysis showed, first and foremost, that participants look at their landscape holistically. As a result, the maps not only reveal a community perspective of dynamics induced by mining but also of shifts between land uses due to changes in agriculture and expanding settlements.

Six observations emerge from the mapped landscapes. First, both study areas experienced an expansion of mining activities from the past to the present. In both, mining is expected to decline, except in Osau, where large-scale mining prevails, suggesting that ASM is a transient land use. Second, while this study started assuming that primarily mining would affect available farming land, settlement expansion was an unexpected factor in determining the decrease in farming land. This suggests a trend toward rural urbanization, often neglected in existing studies (see also Asubonteng et al. 2021, this issue). Third, where mining increases, the settlement area also expanded due to the accommodation needs of immigrant miners, with a combined negative effect on land available for farming. Fourth, while the previous point suggests an anticipated decline in land under food crops, mixed trends resulted from a qualitative assessment of the food-crop coverage (Table S2, supplementary material). Food-crop land is expected to increase in half of the community landscapes and to decrease in the other half, with a decline more often mentioned in the Fanteakwa South District where more permanent large-scale mining occurs. Not only mining plays a role in these trends, but also the expansion of tree crops and settlements, with no strong relationship to a particular land use emerging (see Suppl. Mat.). Fifth, we observed differences between our landscape categorization and the RMSC (2016) typology based on the relative prevalence of farming and mining that we used for the selection of study communities. These differences are due to the different base years when both typologies were developed: in 2018, when the maps of the present were drawn, a ban on small-scale mining was in place. This was not the case when the RMSC made its classification, resulting in a higher prevalence of mining in 2016. Finally, the participatory maps suggest that the landscape has shifted from a generally integrated agricultural landscape in the past to a segregated landscape in the present, with further segregation and homogeneity anticipated for the future. These findings confirm the analysis of satellite images by Asubonteng et al. (2018, 2020), who identified cocoa and oil palm expansion as the main driver at the landscape scale. However, at the more localized level of community areas where mining occurs, landscape actors attribute this to the introduction and expansion of mining activities and associated settlement expansion. These shifts in land use and the fragmentation of farmland where ASM occurs have led to the loss of both food-crop and tree-crop lands.

### The value and risks of participatory mapping

Mapping and assessing changes in rural landscapes from a community perspective is important for two reasons. First, it potentially creates awareness among landscape actors on changes in the landscape and the effects thereof. It was undisputed among the study participants that the landscape is changing from an integrated into a segregated one. In their view, the introduction and expansion of mining and accompanying settlement growth negatively affected the land available for farming, particularly food crops. Of particular concern to them was how water pollution, un-reclaimed mined lands, and forest and biodiversity loss impacted the provision of ecosystem services (see also Rodríguez-Loinaz et al. 2015; Asubonteng et al. 2021, this issue). Participatory mapping and related spatial methodologies also allow landscape actors to learn how their activities and actions impact the landscape, what is needed to curb undesired changes, and their potential role in bringing about the desired change. Such awareness is an essential precondition for a proactive role in landscape governance.

Second, participatory mapping helps landscape actors gain knowledge from the mapping process and the accompanying engagement and discussions with other landscape actors. This can have an empowering effect, as observed in a study by Somuah (2018). As such, it is a valuable tool for inclusive landscape governance, as it allows the involvement of community actors in problem definition and exploring solutions (see also Asubonteng et al. 2021, this issue).

However, words of caution are needed as well. Participatory mapping can also be disempowering, for instance, by creating new power and knowledge disparities between spatial knowledge holders and other community members (e.g., see Anthias 2019) or when particular groups are excluded from the mapping process (e.g., see Pfeffer et al. 2011). This may also be the case if information that could challenge certain privileges of the local elite (male and elderly) is withheld from the map (McCall and Minang 2005: 344). At the same time, mapping resources in the landscape could expose locations for exploitation, which communities prefer to be kept secret (such as sacred sites). Participatory mapping “does not occur in a social vacuum” (Reyes-García et al. 2012, p. 657) and several ethical issues should therefore be considered when applying it (Rambaldi et al., 2006a; see also Somuah et al. 2021, this issue). These include preventing unrealistic expectations and potential conflicts in a community and extracting information that favors outsiders only and can even be used against the community (Chambers 2006). Another limitation of participatory mapping is that the mapped landscape dynamics only reveal changes of interest to the participants. The findings can be made more robust when combined with other methods (Diniz et al. 2015), which was beyond the scope of this paper. Finally, participatory mapping may suffer from a recall bias as far as past dynamics are concerned. This was also a risk in the present study as the past landscape referred to a period when the younger participants were children. We attempted to compensate for this by ensuring that participants were spread over different age groups, including those who had lived long enough in the area to witness changes over three decades. We also enabled all community members to provide inputs in a validation meeting where the produced maps were discussed.

## Conclusions

This study examined how inhabitants of ASM-affected communities in the Eastern Region of Ghana perceive the spatial dynamics in their landscapes and their implications, notably for land available for food production. They visualized this on participatory maps, which revealed the location and spatial arrangement of the main land-cover types in the past (1986), present (2018), and future (2035). These maps revealed a dynamic landscape, changing from a predominantly agricultural landscape to a mining or mine-expanding landscape in the present and a more urbanized landscape in the future. ASM and, to a lesser extent, large-scale mining were the main perceived drivers of landscape change in these purposively selected communities, fueling a loss of food-crop lands and expansion of settlements. Cocoa is expected to become the dominant crop in this farming-mining landscape. Whereas we initially conceived a landscape typology based on the relative dominance of farming and mining, the expansion of settlements and rural urbanization as drivers of landscape change and loss of food-crop land in this area came as a surprise.

No firm conclusions can be drawn about trends for land under food crops. Whereas findings showed that food-crop lands declined in four community landscapes from the past (1986) to the present (2018) due to mining or the expansion of cocoa, this declining trend cannot be extrapolated to the future. Food-crop land is expected to decrease in half of the community landscapes and increase in the other half, with varying relationships to other land-cover types.

Regarding the research approach used, the findings show the potential of participatory mapping to bring landscape actors together to define a common concern entry point (Sayer et al. 2013) and possible solutions. This can contribute to inclusive landscape governance by raising awareness of landscape changes and effects and the need for collective action. Although not resulting from this study due to the homogeneity of the groups engaged in the mapping process, the broader literature indicates that power differences and diverging interests should be considered and that ethical considerations are warranted to prevent elite capture and exclusion of less powerful people. Future research could explore how the perceptions of past and present dynamics compare with results of remote sensing analysis and how anticipations for the future compare to modeling.

## Supplementary information

Supplementary material

## Footnotes

1 Considering the large numer of communities in the area, we feel that the use of base maps does not compromise the desire of the communities to remain anonymous.

2 Taboo days are forms of traditional natural resource governance and informal institutions for the management of natural resources (Colding and Folke 2001; Osei-Tutu 2017).

3 Here and in Figs. 3–7, the scale is based on the base map used for participatory mapping. It should however be noted that the features on the map are not drawn to scale, as is inherent in participatory mapping.

4 Ataa-Asantewaa, M. (forthcoming). Smallholders unpacked: Farmer diversity and engagement in tree-crop value chains in Ghana. PhD thesis, Amsterdam.
