# Supplementary material for: Using Participatory Spatial Tools to Unravel Community Perceptions of Land-Use Dynamics in a Mine-Expanding Landscape in Ghana
Source: Environ Manage. 2021 Jul 1;68(5):720–37. doi: 10.1007/s00267-021-01494-7 (PMC8560661; doi:10.1007/s00267-021-01494-7)

## Supplementary material

## Assessing Trends and Trade-Offs between Farming, Mining, and Settlement

To analyze the trends in the coverage of the major-land cover types – farming, mining, and settlement − we designed a scoring scheme (Table S1). The first assessment was done by each author individually, based on agreed percentages to quantify coverage for each land-cover type on a six-point scale from zero (0) to very high (5). Whereas zero means ‘Not occurring’ in all cases, the proportions chosen to distinguish between the range from ‘very low’ to ‘very high’ occurrence were different for farming and the other two land-cover types, based on the authors’ perceptions of what these qualifications mean for the three land-cover types in a traditional rural landscape in the region (Table S1).

When estimates differed among the authors, four quadrants were drawn on each map, after which the proportional coverage of each land-cover type was re-assessed per quadrant. The estimates of land-cover types per quadrant were summed and then divided by four. The resultant percentages were then matched with the scores in Table S1 for a final assessment of the occurrence of each land-cover type. These were subsequently presented in radar diagrams to discern the trends reported in the results section.

**Table S1** Scoring scheme for each of the major land-cover types

| Score | Occurrence | Explanation |
| --- | --- | --- |
| 0 | Not occurring | Absent in the landscape map |
| 1 | Very low | Farming < 20%; mining <10%; settlement <10% |
| 2 | Low | Farming 20-40%; mining 10-20%; settlement 10- 20% |
| 3 | Moderate | Farming 40-60%; mining 20-40%; settlement 20-40% |
| 4 | High | Farming 60-80%; mining 40-60%; settlement 40-60% |
| 5 | Very high | Farming >80%; mining >60%; settlement >60% |

Trends in the relative abundance of food crops related to other land-cover types across time (Table S2) were assessed through visual observation of the maps. Trends for land under farming, mining, and settlement are based on the scores in the radar diagrams (Figures S1-6).

From 1986 to the present, the area under food crops had declined in three community landscapes, all but one with a high prevalence of mining (Makisa, Gyesame, Osau).

‘This community was a food basket and because of this there was this common saying in our community ‘if the people in the big towns have eaten to their fill, then our community can enjoy peace’. But now this saying is no longer heard because most of our lands are under mining or have been mine and we can hardly produce enough for ourselves to export the surplus.’ (Workshop participant Makisa, June 2019).

In the case of Gyesame, the decrease went together with a high prevalence of farming and is mainly to be attributed to the expansion of cocoa (Fig. 2a-b). The declining trend in food-crop areas is reversed where mining disappears from the future landscape, even when settlement area scores moderate to very high (Makisa, Gyesame). However, food crop areas are expected to decline in the future where mining continues and is combined with high settlement cover (Mudawka, Wanoiso, Nanaase). Osau is a seeming outlier, with an expected increase in food-crop land despite a score of 4 for mining and moderate settlement area in the future.

**Table S2** Trends regarding land under food crops relative to other land-cover types

| Community landscape | 1986-2018 | | | | 2018-2035 | | | |  |
| --- | --- | --- | --- | --- | --- | --- | --- | --- | --- |
|  | Farming | Mining | Settle-ment | Food crops | Farming | Mining | Settle-ment | Food crops | |
| Gyesame | - | + | ± | - | ± | - | + | + | |
| Makisa | - | ± | + | - | + | - | + | + | |
| Mudawka | + | + | + | + | - | - | + | - | |
| Nanaase | - | + | + | ± | + | - | + | - | |
| Wanoiso | - | + | + | ± | ± | - | + | - | |
| Osau | - | + | + | - | - | ± | + | + | |

+ increased; ± remained the same; - decreased.


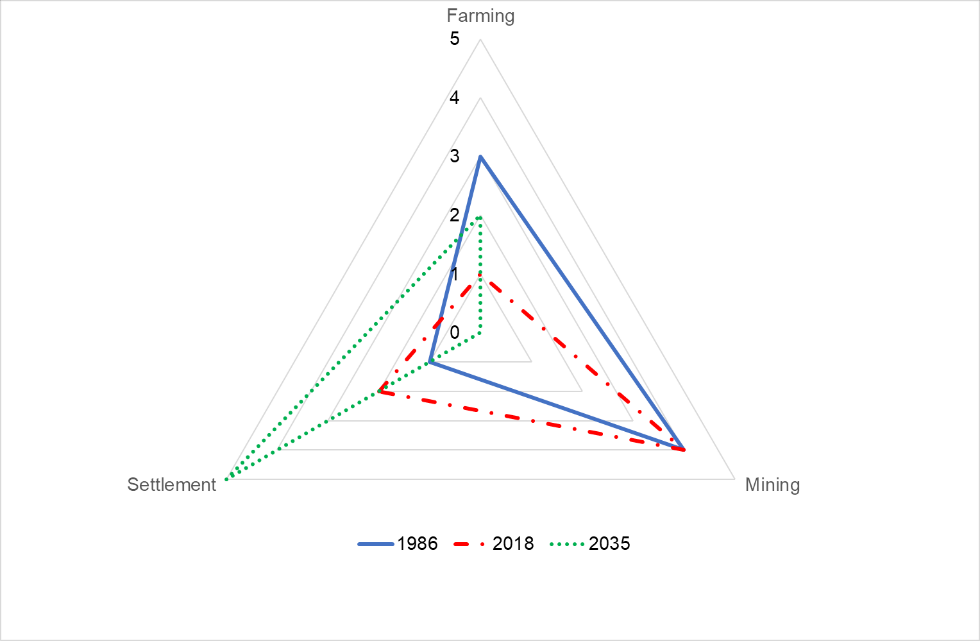


**Fig. S2** Radar diagram showing trends in the Makisa landscape


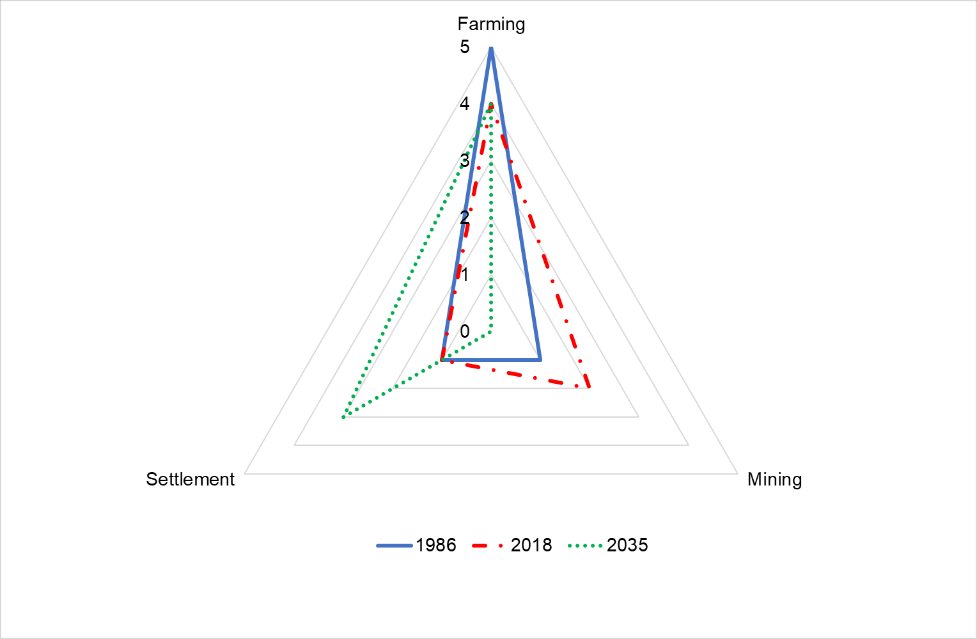


**Fig. S1** Radar diagram showing trends in the Gyesame landscape


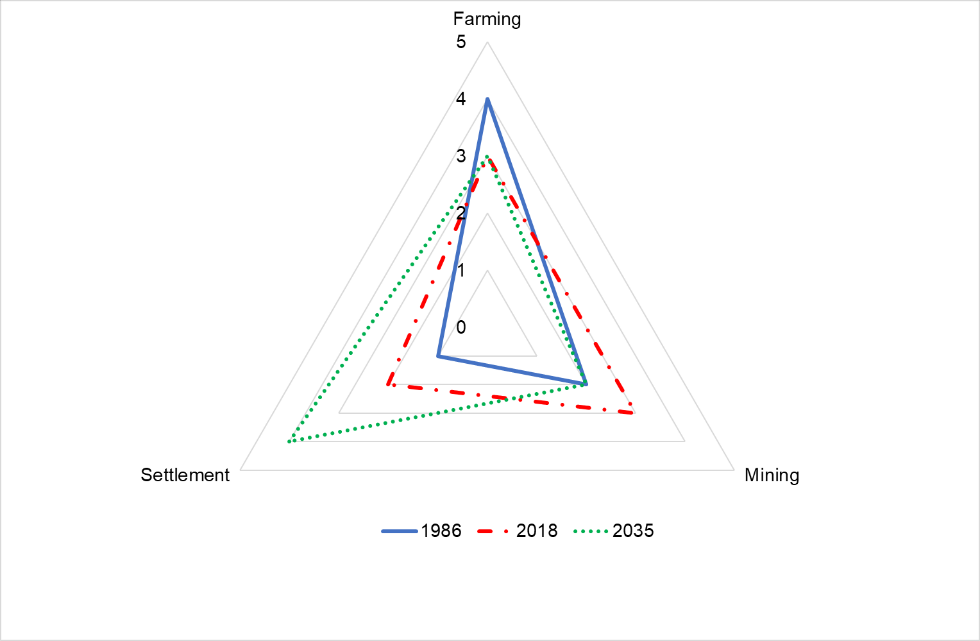


**Fig. S5** Radar diagram showing trends in the Wainoso landscape

**Fig. S6** Radar diagram showing trends in the Osau landscape


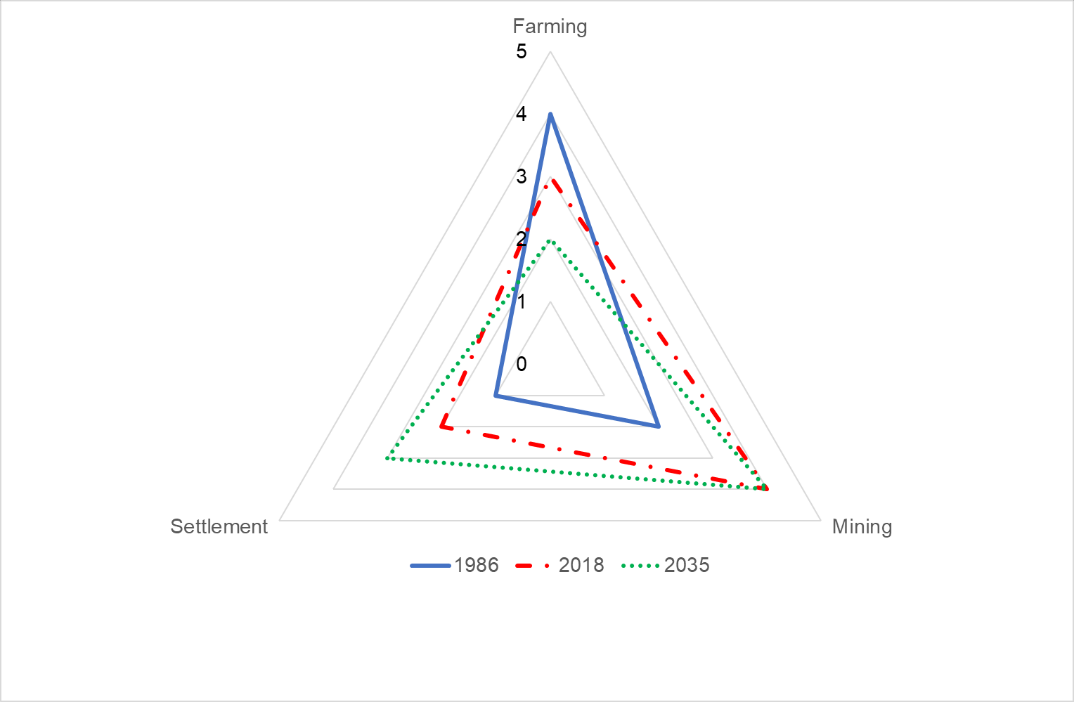


**Fig. S3** Radar diagram showing trends in the Mudawka landscape


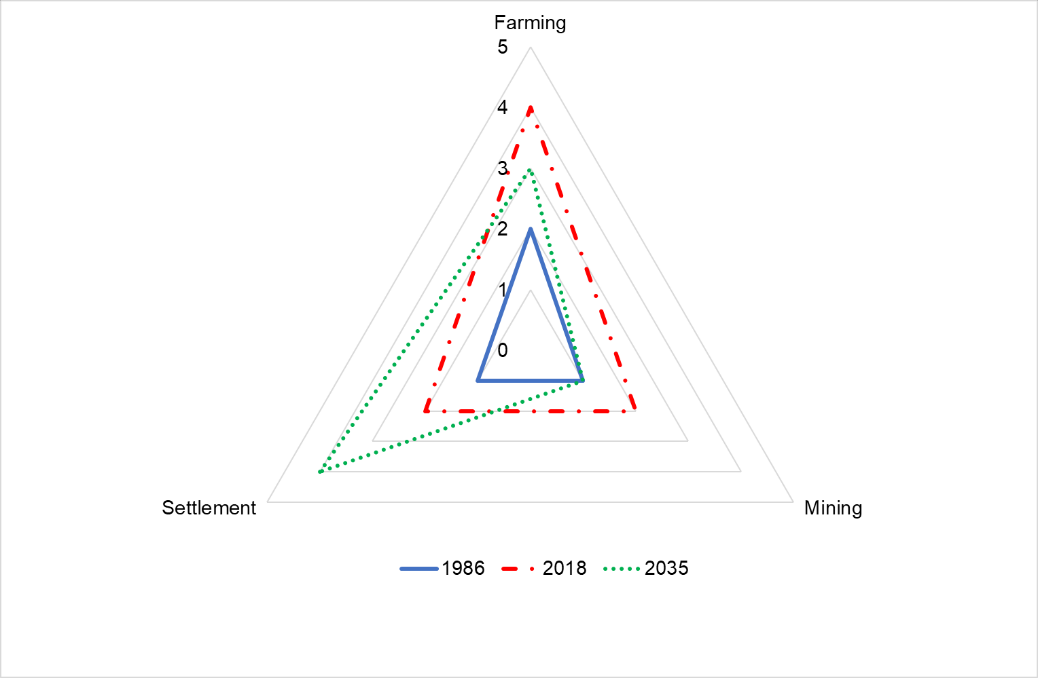


**Fig. S4** Radar diagram showing trends in the Nanaase landscape


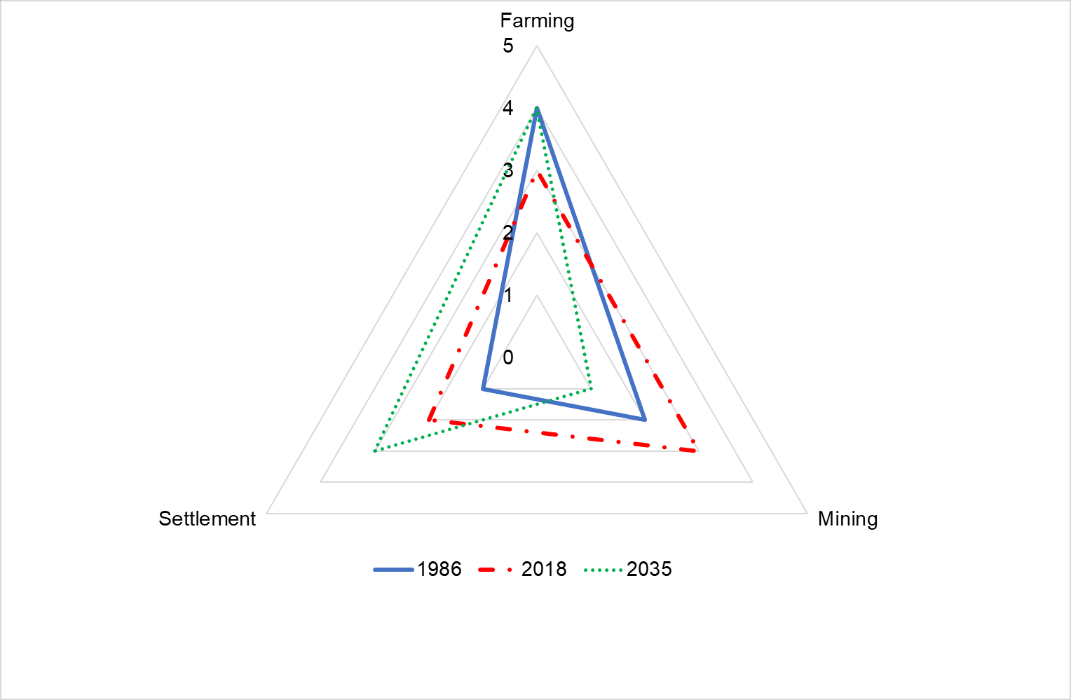

Supplement: Supplementary file 1 — Supplementary material [file 267_2021_1494_MOESM1_ESM.docx]
